# Trends in sports participation in adolescents: Data from a large-scale sample in the US adolescents

**DOI:** 10.3389/fpubh.2022.960098

**Published:** 2022-10-31

**Authors:** Yu Deng, Anhui Fan

**Affiliations:** Department of Physical Education, Southwest University Chongqing, Chongqing, China

**Keywords:** sport participation, adolescents, US, physical activity, trends

## Abstract

**Background:**

Although changes in overall physical activity (PA) have been identified in adolescents, the trend in sports participation is still understudied. It is widely believed that monitoring the changes in sports participation is conducive to promote the development of sports activities. The purpose of this research was to identify the changes in sports participation in adolescents over the past years.

**Methods:**

This research selected secondary data from 2011, 2013, 2015, 2017, and 2019 Youth Risk Behavior Surveillance (YRBS). Logistic regression models were applied to discuss and analyze the secular changes across the years of data. The change differences in sports participation by sex, grade, and race/ethnicity were also explored *via* separate logistic regression.

**Results:**

A declined overall trend could be observed in sports participation in adolescents, the prevalence of sport participation was 58.4% in 2011 and 57.4% in 2019. The declining trend was also observed in grades 10 (62.3% in 2011 and 57.9% in 2019) and 12 (52.5% in 2011 and 49.8% in 2019) adolescents, and an increase could be observed in grade 11 (56.2% in 2011 and 59.1% in 2019) adolescents, but few changes were found in grade 9 (61.4% in 2011 and 61.9% in 2019) adolescents. Only white adolescents reported an increasing prevalence of sports participation, slight declines in sports participation were observed in black or African American, Hispanic/Latino, and other adolescents.

**Conclusions:**

The declining trend in sports participation could be seen in adolescents between 2011 and 2019, but it should also note that large variations of trends in sport participation by subgroups were also found.

## Introduction

Sports participation is the outcome of basic education reform and represents a new field of learning (1). Sports participation is not only a new overall goal of education for students but also an important way to realize children's socialization (2, 3). The word “participation” is bound up with management and organizational behavior, and it is the basis to measure whether an individual, as an entity, participates in activities (4). However, with the emphasis on the development needs and inner psychology of students, the research began to highlight the involvement in cognitive and emotional aspects and gradually explored the external factors affecting sports participation of teenagers (5). Sports participation means mental and physical energy input of teenagers (6). The initiative degree of students' sports participation can be judged not only by external indicators that include heart rate, expression, and emotion but also by students' attitude and persistence (7).

Ren et al. (8) investigated over 7,000 students to model the relationship between family and community resources and the frequency of sports participation of students and found that only one-sixth of the students participated in sports clubs or planned sports by themselves, so they classified most of the students into the sub-health group and found that in the sub-health group, girls, senior students, and Han people accounted for a large proportion (8). It has been known that family culture has a strong promotion effect on sports participation (9). By conducting structured interviews with sports children, parents can set scientific goals and dietary collocation for them to better promote their children to get a pleasant experience of sports and persist in it (10). However, these studies on the relationship between sports participation and family communities were selected from a specific region or interviewed in small numbers, resulting in limiting other regions that represent different education policies.

At the same time, Chen et al. (3) focused on the factors affecting youth sports participation. Based on the data provided by Youth Risk Behavior Survey (YRBS), gender, grade, and academic achievements of American students in grades 9–12 were taken as variables, and it was concluded that academic achievements were positively associated with sports participation. By making a comparison between sports participation and other behaviors of adolescents, the study found that sports participation was associated with many positive health behaviors and the proportion of bad habits is small (11, 12). From the perspective of psychological influence, teenagers' sports participation plays a pro-social role (13). Teenagers enjoy healthy emotions brought by sports and entertainment, which to some extent refutes the connection between sports participation and social resources (8, 14), suggesting that schools should organize sports groups and competitions to intervene in academic achievements of students and different grades adopt diverse sports based on physical characteristics of adolescents. Using nationally representative data from the United States, these reports provide evidence of the relationship between diversified factors and sports participation. However, the specific causes of rank and gender should be further explored.

To our knowledge, only a limited number of studies surveyed changes in sports participation in children and adolescents, despite much evidence on changes in overall PA. Monitoring and forecasting the changes in sports participation enable researchers and practitioners to do effective action plans for encouraging engagement in sports activities by considering further health promotion.

This research targeted reporting changes in sports participation of children and adolescents over the past years. Beyond that, to detect secular changes, we also explored sociodemographic factors related to sports participation.

## Methods

### Study design and population

This study used data from five cycles of the YRBS (2011, 2013, 2015, 2017, and 2019). The YRBS is a biennial, cross-sectional school-based survey of health-related behaviors among a nationally representative sample of high school students living in the United States. The YRBS uses a three-stage cluster sample design to recruit students attending public and private schools in grades 9–12. Students in grades 9–12 in public and private schools in the United States were included in the sampling frame. In the first stage, the primary sampling units (PSUs) were included from counties and adjacent counties. In the second stage, the public and private schools with 9–12 grades were selected from PSUs. In the third stage, one or two entire classes in each grade were randomly selected from the chosen school. The survey was administered in person by trained data collectors and completed by students during class time. Overall response rates were above 60% during the administration of each cycle of the YRBS. Survey results were weighted to represent the populational and national health data. The data used in this secondary analysis were de-identified and publicly available, which have been approved by Centers for Disease Control and Prevention's (CDC) institutional review board. Additional details about the YRBS can be found at https://www.cdc.gov/healthyyouth/data/yrbs/index.htm.

### Measures

Participants provided demographic information about their sex (female/male), grades (9, 10, 11, and 12), and race/ethnicity (white, black/African American, Hispanic/Latino, other). The term “sports participation” refers to playing on 1 or more sports teams during the past 12 months (15). Sports participation was assessed by one single question, which was “during the past 12 months, how many sports teams did you play? (Count any teams run by your school or community groups.)” Responses to the question included 0 teams, 1 team, 2 teams, and 3 or more teams. This single question has been reported to be reliable for measuring sport participation in a previous study (15, 16).

### Statistical analysis

All the variables included in this study were treated as categorical variables. For each variable, weighted prevalence estimates with 95% confidence intervals (CIs) were calculated while accounting for complex sampling surveys, using Taylor linearization to produce nationally representative prevalence estimates for each survey year. To examine trends in sport participation and across the 2011–2019 cycles of the YRBS, logistic regression models were conducted with time-trend variables that assess secular changes across the years of data. Separate logistic regression models were also performed to explore associations between sociodemographic variables (sex, grade, and race/ethnicity) and sport participation, which generated year-based and year-combined associations. Adjusted odds ratio (OR) with 95% CI after controlling for sex, grade, and race/ethnicity are presented for all logistic regression models. All analyses were performed using SVY procedures by taking sampling stratum, primary sampling unit, and weight based on the YRBS protocol in Stata/IC 16.1 (Stata Corp LLC). Statistical significance was considered at a 2-tailed *p* < 0.05.

## Results

**Figure 1:**
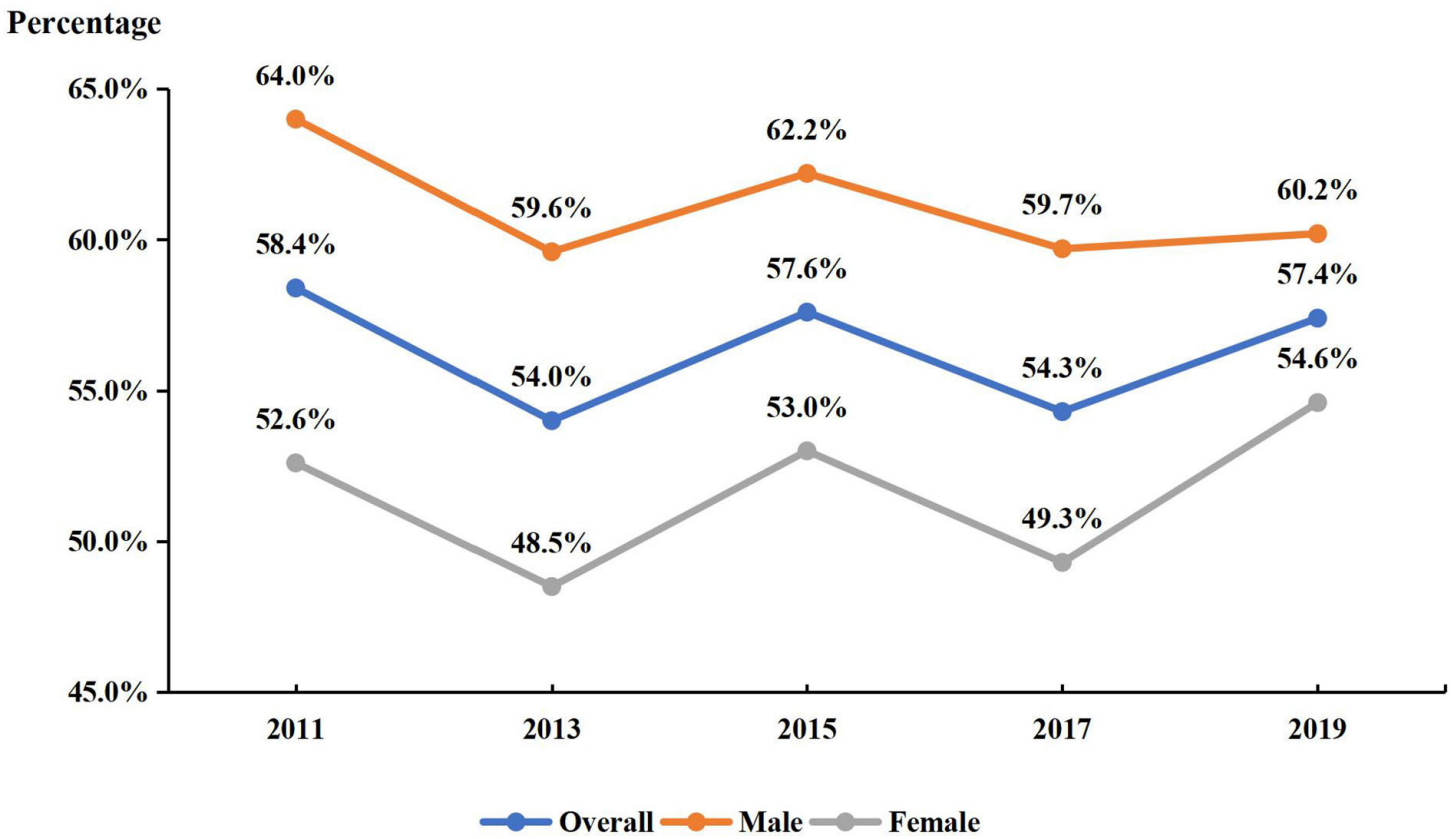
Prevalence of participating in at least one sports team over the past 12 months in the sample (overall and by sex) between 2011 and 2019. For the overall sample, models were controlled for sex, grade, and race/ethnicity. For sample by sex, models were controlled for grade and ethnicity.

The demographic characteristics of participants are shown in Table 1. In total, 13,677 (50.3% girls) adolescents were recruited to participate in this survey. The weighted percentage of female participants was 49.4%, more than half of the participants were white, and 47.9% of students were over grade 11. The overall sex prevalence of participating in sports teams by each year is outlined in Figure 1. It can be seen from Figure 1 that the prevalence of participating in boys experienced a declining trend (from 64.0 to 60.2%), while the girls witnessed a slight rising trend between the years 2011 and 2019 (52.6–54.6%). In the overall sample, after trend analysis, there was statistically significant declining changes in sport participation (both linear and quadratic, *p* < 0.005). Similar significant changes were also observed in only boys (both linear and quadratic, *p* < 0.001) instead of girls (*p* > 0.05).

**Table 1:** Demographic characteristics of the participants.

		***n***	**%**	**Weighted %**	**95%CI**
Total		13,677	100	/	/	/
Sex						
	Female	6,885	50.3	49.4	47.9	50.9
	Male	6,641	48.6	50.6	49.1	52.1
	Missing	151	1.1			
Grade						
	9th	3,637	26.6	26.6	25.4	28.0
	10th	3,717	27.2	25.5	24.7	26.3
	11th	3,322	24.3	24.3	23.2	25.4
	12th	2,850	20.8	23.6	22.4	24.8
	Missing	151.0	1.1			
Race						
	White	6,668	48.8	51.2	46.4	56.0
	African American	2,040	14.9	12.2	10.2	14.6
	Hispanic/Latino	3,038	22.2	26.1	21.8	30.9
	All other races	1493	10.9	10.5	7.9	13.9
	Missing	438	3.2			

CI, confidence interval.

**Figure 2:**
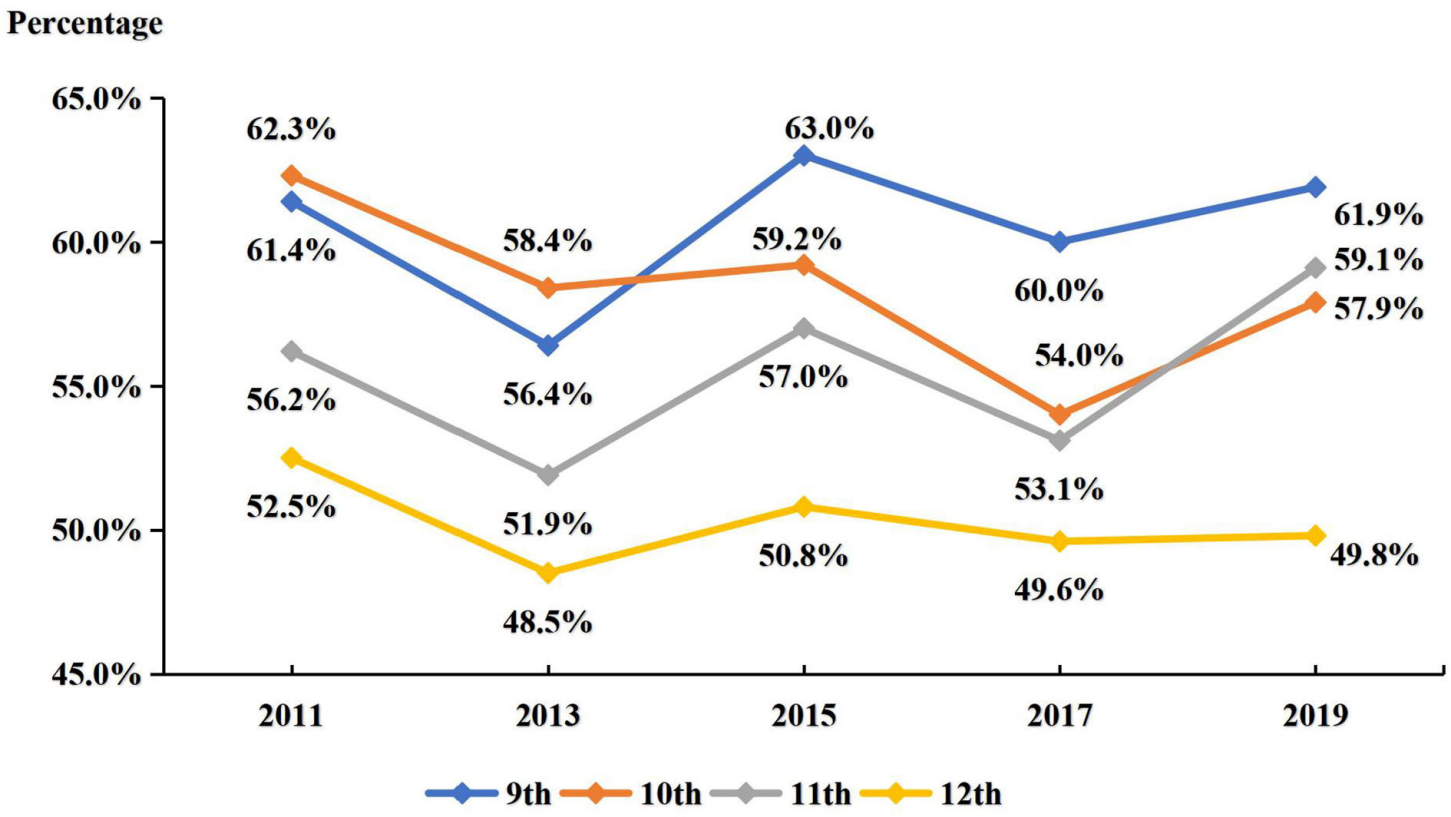
Prevalence of participating in at least one sports team over the past 12 months in the sample (by grade) between 2011 and 2019. All models were controlled for sex and race/ethnicity.

Secondly, by analyzing grade parameters (shown in Figure 2), compared grades 10 and 12 experienced a downward trend and grades 9 and 11 demonstrated a rising trend from the year 2011 to 2019. The trends of sport participation over the past 10 years are presented in Figure 2. In grade 9, no significant changes were observed (*p* = 0.129). Similar changes were observed in grade 11 students. Significant quadratic declining trend of sport participation was detected in grade 10 students (*p* = 0.009) and grade 12 students (*p* = 0.038).

**Figure 3:**
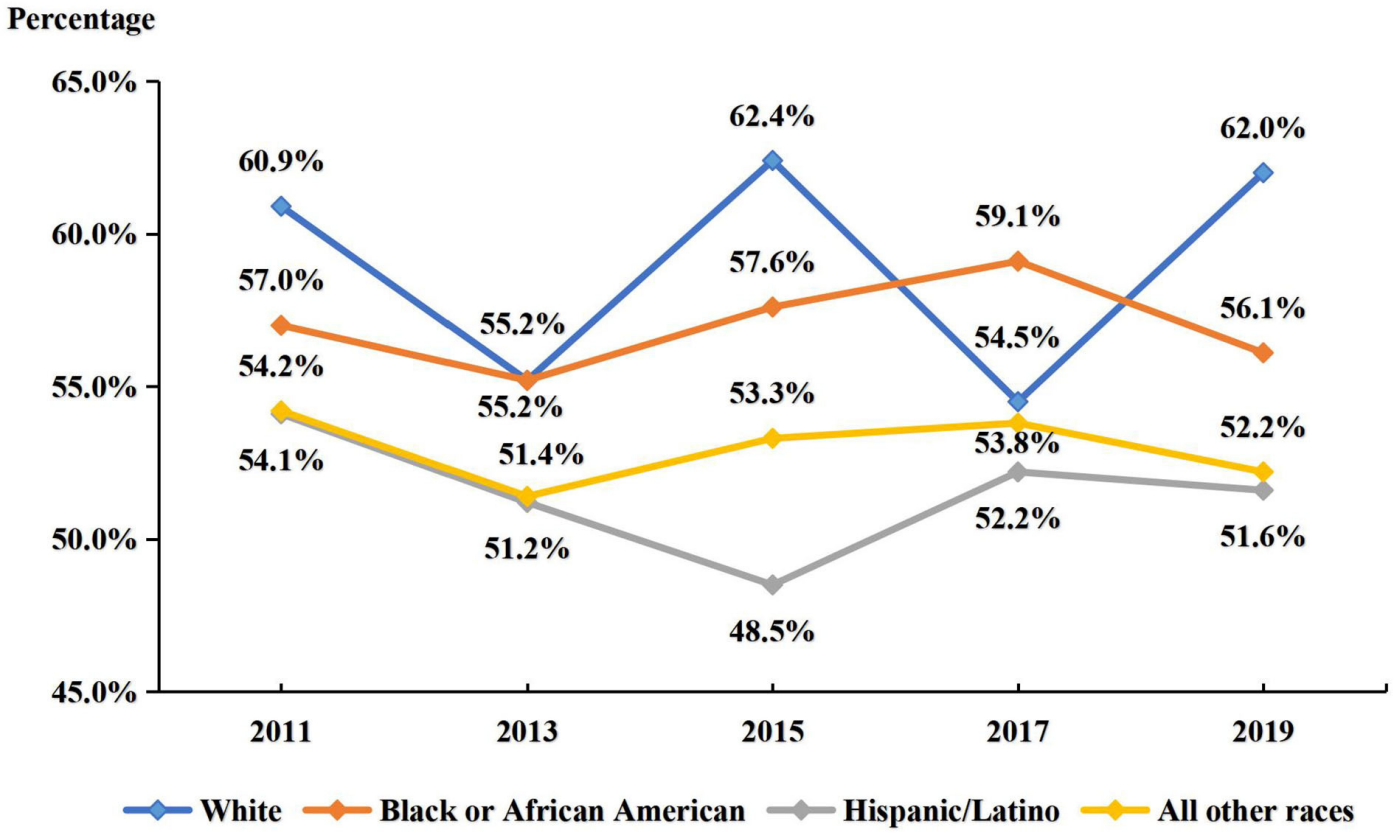
Prevalence of participating in at least one sports team over the past 12 months in the sample (by race/ethnicity) between 2011 and 2019. All models were controlled for sex and grade.

The prevalence of participating in at least one sports team by race/ethnicity is shown in Figure 3. Analysis by race/ethnicity showed that white adolescents had the highest sports participation, only surpassed by black or African American adolescents by 4.6% in 2017 and about the same percentage as other races and Hispanics/Latinos. In 2019, however, sport participation outnumbered blacks by about 6% and Hispanic/Latino or other races by about 10%. The trend analysis revealed different trends of sport participation in adolescents of different races/ethnicities. In white adolescents, no significant changes were found (*p* > 0.05). In black or African American adolescents and those of all other races, no significant changes were also observed (*p* > 0.05). However, in Hispanic/Latino adolescents, a significant quadratic declining trend was found (*p* < 0.05).

Table 2 demonstrates results for the relationship between sociodemographic factors and sports participation. It can be seen from Table 2 that relative to girls, boys show greater odds of engaging in sports participation (OR = 1.50, 95% CI: 1.40–1.61). In addition, the odds for sports participation in 9th graders (OR = 1.53, 95% CI: 1.41–1.67) were significantly higher than those in 12th graders. When it comes to ethnicity, people from white were more likely to get involved in sports participation (OR = 1.10, 95% CI: 0.99–1.23) than black or African adolescents.

**Table 2:** Odds ratio and 95% CI for socioeconomic factors concerning sports participation in this study.

	**OR**	**95%CI**	
Male	1.50	1.40	1.61
Female		REF
9th	1.53	1.41	1.67
10th	1.41	1.32	1.51
11th	1.23	1.15	1.32
12th		REF
White	1.10	0.99	1.23
Hispanic/Latino	0.80	0.73	0.87
All other races	0.86	0.79	0.95
Black or African		REF

OR, odds ratio; CI, confidence interval; REF, reference group.

## Discussion

The purpose of this research was to investigate the trend of sports participation in American adolescents from 2011 to 2019 and the sociodemographic factors of sport participation. Evident changes in overall sports participation were observed in adolescents, but with large variations of the trends in adolescents by subgroups (e.g., boys were different from that of girls). Regarding the factors influencing sport participation, boys are lower graders were two important and positive factors of sport participation. More analysis is presented below.

Our study confirmed that boys were more possible to participate in sports than girls, which can be supported by previous studies (3, 8, 17), For instance, there was a study by the YRBS that found that girls were less likely to participate in physical activities than boys, which indicated that girls might encounter huge barriers to take part in sports activities (17). Previous studies indicated that ratio of sports participation of girls was inferior to that of boys (18). Within the restricted sports environment or resources, boys were able to control the space and facilities of sports activities while girls might be isolated or excluded (19). Furthermore, there is evidence indicating that support of parents might be an important explanation because fathers tend to participate in the sports activities of their sons more than their daughters'. In this way, it is essential to safeguard resources and chances of sports participation in the school environment. Beyond that, given sex stereotypes and different social roles (20), boys performed more possibilities to attend sports activities while girls preferred to attend more leisure activities and personal art activities (17). Another factor should be puberty and menarche, such as girls entering early into puberty than boys (21). As a result, it is of great significance to construct positive female images under the sports content in which existing stereotypes and inspired sports participation (especially for girls) will be disadvantaged to some extent.

This research suggested that an overall decline in overall sports participation was observed in adolescents between 2011 and 2019, which was inconsistent with two previous studies (22, 23). For example, a stable increasing trend of sports participation was seen in Icelandic adolescents (24). However, it also should be noted that more than half of adolescents did not consistently participate in organized club sports (25). In addition, another study also reported a similar finding that there was an increasing trend of sports participation in Sweden adolescents (26). These two studies are inconsistent with our research findings. Several explanations might contribute to the difference between the current research and the previous studies, such as the sports opportunities, facilities, and sports policies (22). As the current study merely reported secular trends of sport participation over the past years in adolescents, there was no other information on better understanding or further explaining the trends, future studies based on the contexts and backgrounds should be put forward for reversing this declining trend.

When looking at the trends of sports participation in adolescents by groups (i.e., sex, grade, and race/ethnicity), some notable and interesting research findings are worth mentioning. First, in boys, although there was an overall decrease in sport participation over the past years, its changes underwent an evident variation. For example, from 2011 to 2013, an apparent decrease in sport participation was observed, following an increase by 2015. After that, a general but slight decrease was observed. Unlike this, in girls, a general but non-significant increase in sport participation was found, but also with some fluctuations from 2013 to 2017. The sex discrepancy that resulted in different trends in sport participation in boys and girls might be owing to sex-specific perceptions toward and engagements in sports activities in different survey years. In the current study, owing to limited data collected (public data), we cannot provide further insight into the sex difference, which should be addressed in the future. In terms of grade difference, graders 9 and 11 have no significant changes in sports participation, probably because of great variations in different survey years. Conversely, in graders 10 and 12, decreases in sport participation over the past years were found. It is expected that higher grade students should have sharper decreases in sport participation, but this study did not find such research findings. Unfortunately, we cannot consider the contexts of different survey years, such as specific survey time, sample characteristics, and other factors. In the future, to explore the variations of trends in sport participation in adolescents by grade well, it is needed to know more relevant contextual information. Regarding adolescents by race/ethnicity, only adolescents of Hispanic/Latino had a significant decreasing trend in sport participation, but adolescents of other races/ethnicities were not found with decreasing trend. This research finding indicates that the decreasing trends of sport participation in Hispanic/Latino adolescents should be considered when designing interventions. The present study extends the literature by identifying more nuanced patterns of changes in sport participation. Of note, however, it should be extremely cautious to compare our research results with the previous studies because of diversely cultural differences and contextual factors. To our knowledge, there is no comparable sport participation research for the past decade on the possible differences in sex-stratified, grade-stratified, and race/ethnicity-stratified groups; so that such novel research findings may highlight adolescents from different subgroups are undergoing health behavior disparities. This would be an evidence base for tailoring-specific measures aimed at increasing participation in sports activities.

Although this research analyzed a large sample to identify the trends in sports participation by sex, grades, and race/ethnicity in adolescents, several limitations should be addressed. First of all, this research made use of cross-sectional data from YRBS. In addition, adolescents were required to self-report their sports participation over the past 12 months, and the self-administrated questionnaire might contribute to recall bias and lead to underestimates or overestimates of the level of sports participation.

## Conclusion

To sum up, data from YRBS reported an overall declining trend in sports participation in United States adolescents, but the trends varied greatly by different subgroups (e.g., sex and grade). Future studies should further explore the trends of sport participation in adolescents and design effective strategies to promote this population to engage in more sports activities in terms of health promotion. To promote sports participation in adolescents, girls and older adolescents (higher graders) are the target priority.

## Data availability statement

The original contributions presented in the study are included in the article/supplementary material, further inquiries can be directed to the corresponding author/s.

## Ethics statement

The studies involving human participants were reviewed and approved by all data were anonymized and publicly available; therefore no ethical approval was required. Written informed consent to participate in this study was provided by the participants' legal guardian/next of kin.

## Author contributions

YD: writing—original draft. YD and AF: formal analysis and writing—review and editing. Both authors contributed to the article and approved the submitted version.

## Funding

This study was supported by the 2021 Tianjin Education Commission Scientific Research Program Project: Research on the Exploration and Application of Sports Rehabilitation Combined with Acupuncture and Physical Therapy in the Treatment of Low Back Pain after Delivery of Pregnant Women (research project number: 2021KJ067).

## Conflict of interest

The authors declare that the research was conducted in the absence of any commercial or financial relationships that could be construed as a potential conflict of interest.

## Publisher's note

All claims expressed in this article are solely those of the authors and do not necessarily represent those of their affiliated organizations, or those of the publisher, the editors and the reviewers. Any product that may be evaluated in this article, or claim that may be made by its manufacturer, is not guaranteed or endorsed by the publisher.
